# Green tea increases the survival yield of Bifidobacteria in simulated gastrointestinal environment and during refrigerated conditions

**DOI:** 10.1186/1752-153X-6-61

**Published:** 2012-06-22

**Authors:** Dan C Vodnar, Carmen Socaciu

**Affiliations:** 1 Food Science and Technology Department, Unit of Chemistry and Biochemistry, University of Agricultural Sciences and Veterinary Medicine, 3-5 Mănăştur str, Cluj-Napoca, 400372, România

**Keywords:** Green Tea, *B. Infantis*, *B. Breve*, Microencapsulation, Gastrointestinal conditions, Polyphenols

## Abstract

**Background:**

The well–known prebiotics are carbohydrates but their effects may not always be beneficial, as they can also encourage the growth of non-probiotic bacteria such as *Eubacterium biforme* and *Clostridium perfringens*. Therefore, new alternatives such as non-carbohydrate sources to stimulate the growth of probiotics are needed. The aim of this work was to evaluate (I) the green tea polyphenols by HPLC-LC/MS and (II) the protective effect of green tea extract on viability and stability of *B. infantis ATCC 15697* and *B. breve* ATCC 15700 microencapsulated in chitosan coated alginate microcapsules during exposure to simulated gastrointestinal conditions and refrigerated storage.

**Results:**

The major compound identified by HPLC-LC/MS in green tea was epigallocatechin gallate followed by caffeine and epigallocatechin. The survival yield of probiotic bacteria in microcapsules with 10% GT during storage at 4°C, demonstrated significantly (P < 0.05) higher number of survival bacteria. Microencapsulated *B.infantis* and *B. breve* with 5% and 10% GT showed a significantly (P < 0.05) improved survival under simulated gastric conditions (pH 2.0, 2 h) and bile solution (3%, 2 h) when they were compared with microencapsulation without GT addition.

**Conclusions:**

The results of this study suggest that green tea coencapsulated with *B. infantis* or *B. breve* exert a protective effect of bacteria during exposure to gastrointestinal conditions and refrigerated storage. For a health perspective, the results confirm the growing interest probiotic bacteria and the perceived benefit of increasing their numbers in the gastrointestinal tract by microencapsulation.

## Background

The active delivery of probiotic cells in microencapsulated form has received reasonable attention during the last 10 years, since it can reduce losses of sensitive bacteria induced by detrimental external factors during storage and digestion [1]. Several reviews [2] summarized the potential of microencapsulation to improve probiotic survival during storage or gastrointestinal transit. Alginate is the most widely used matrix for microencapsulation, but its use is limited due to low stability in acidic conditions [3,4]. Therefore coating of alginate with chitosan improved the stability of alginate beads and thus improved the viability of the encapsulated probiotic bacteria, as previously shown by Krasaekoopt et al. [5].

Bifidobacteria selectively colonize the intestinal tract of breastfed infants and are also relevant colonic bacteria in adults [6]. The strains commonly regarded as human probiotics belong to the species *Bifidobacterium bifidumB. breveB. infantisB longumB. lactis* and *B. animalis*, which are included in functional dairy products [6]. *Bifidobacterium longumB. adolescentis*, and *B. catenulatum* are most commonly found in adult faecal samples while *B. infantis* and *B. breve* are predominantly present in infant’s faeces [7]. Though, *B. infantis* and *B. breve* are also found in certain numbers in adults [8]. Strains of *B. infantis* are considered particularly beneficial due to its enhanced ability to inhibit gastrointestinal pathogens through direct anti-microbial action and to attenuate colitis [9].

Prebiotics are non-digestible carbohydrates that beneficially affect the host after ingestion as they are available as a selective energy source for probiotic Lactobacilli and Bifidobacteria, stimulating their growth and activity in the colon [10]. The effects of carbohydrate-type prebiotics may not always be beneficial, as they can also encourage the growth of non-probiotic bacteria. Bello et al. [11] demonstrated that the use of fructo-oligosacharides (FOS) resulted in enhanced growth of *Eubacterium biforme* and *Clostridium perfringens*. Therefore, new alternatives such as non-carbohydrate sources to stimulate the growth of probiotics are needed.

It is believed that the efficiency of probiotic health benefits can be enhanced by coupling the application of probiotics with a selective prebiotic growth substance, thus favoring colonization of probiotics in the human gut [12]. Hence, a combined application of pro- and prebiotics, a concept referred to as symbiotic, also emerged in the field of probiotic microencapsulation.

Green tea (*Camelia sinensis*) is one of the most widely consumed beverages in the world and has multiple health benefits, such as anti-stress [13], anticancer [14], antioxidant [15] and neuroprotective effects [16]. Green tea active polyphenols include (−)-epigallocatechin-3-gallate (EGCG), (−)-epigallocatechin (EGC), (−)-epicatechin-3-gallate (ECG), and (−)-epicatechin [17].

In the present study, we evaluated (I) the green tea polyphenols by HPLC-MS and (II) the protective effect of green tea extract on viability and stability of *B. infantis* and *B. breve* microencapsulated in chitosan coated alginate microcapsules during exposure to simulated gastrointestinal conditions and refrigerated storage.

## Results and discussion

### HPLC-LC/MS characterization of green tea extract

**Figure 1:**
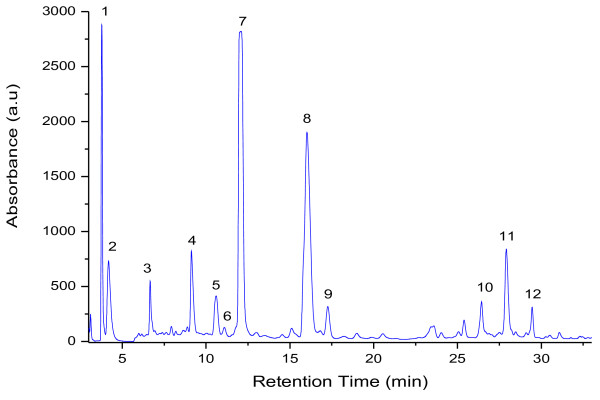
**HPLC chromatogram of phenolic compounds in green tea extract.** Peak numbers correspond to Table1.

Figure 1. shows a chromatogram of extracted green tea while the Table 1 lists the retention times, mass data, concentration and the substance names for the numbered peaks in the chromatogram. The compounds in tea infusion were identified with reference compounds and literature data on the basis of their HPLC retention times and mass spectra.

**Table 1:** Retention time, mass spectral data and concentration of phenolic compounds from green tea extract

**Peak nr.**	**R**_**t**_**(min)**	**[M + H]**^**+**^***(m/z)***	**Compound**	**Concentration mg/ml**
1	3.77	335	Galloylquinic acid	6.18
2	4.17	171	Galic acid	0.59
3	6.66	307	Gallocatechin	4.5
4	9.13	307	Epigallocatechin	7.13
5	10.60	340	Dicafeic acid	0.32
6	11.09	291	Catechin	1.59
7	12.08	195	Caffeine	19.16
8	16.02	291	Epicatechin	3.34
9	17.26	459	Epigallocatechingalate	53.18
10	26.42	304	Ellagic acid	0.82
11	27.91	443	Catechingallate	3.29
12	29.45	466	Quercetin glucoside	0.35

**Figure 2:**
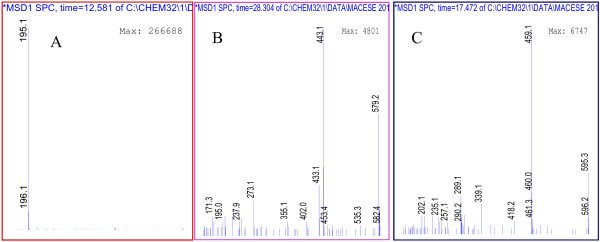
**Mass spectra of Caffeine 195*(m/z)*(A), Catechingallate 443*(m/z)*(B), Epigallocatechingalate 459*(m/z)*(C).**

The dominant peak 7 in Figure 1, was identified as caffeine by processing the same retention time and mass spectrum, which had a [M + H]^+^ ion at *m/z* 195 Figure 2A. Peak 3, 4, 6, 8, 9, 11 were all identified as catechins belonging to the flavan-3-ol class of flavonoids. Peak 3 and 4 had a [M + H]^+^ ion at *m/z* 307 and according to the mass fragment ions were identified gallocatechin and epigallocatechin. Peak 6 was identified as catechin which had a [M + H]^+^ ion at *m/z* 291. Peak 9 showed a mass spectrum which corresponded with epigallocatechingalate Figure 2 C. Peak 11 had a [M + H]^+^ ion at *m/z* 443 (Figure 2B) which clearly confirmed the catechingallate compound. Peak 1 and 2 were identified as phenolic acids according to the mass spectrum. Peak 1 produced the [M + H]^+^ ion at 335 and mass fragment ions at *m/z* 189 and 171, which corresponded to quinic and gallic acid. Thus, peak 1 was identified as galloylquinic acid, peak 2 was confirmed as gallic acid. As can be seen in Table 1, the major compound in green tea extract was epigallocatechingalate 53.18 mg/ml followed by caffeine 19.16 mg/ml and epigallocatechin 7.13 mg/ml.

### Microcapsules characteristics. Size, entrapment efficiency and viability

Table 2. shows results for diameters and encapsulation yields of chitosan-coated alginate microcapsules containing *B. infantis* or *B. breve* with or without addition of green tea. The mean diameters of all types of microcapsules were between 318.23 and 344.19 μm. *EY* was higher for microcapsules with *B. infantis* as compared to all trials and low differences between microcapsules with *B. breve*, B + 5%GT, I + 10% GT. Some studies reported that encapsulation of probiotic bacteria with quercetin (prebiotic) has poor encapsulation efficiency and low viability of the cells in quercetin beads due to interaction of flavonoid with probiotic [18,19]. Our results showed difference between viability of bacteria in beads, increasing the viability of bacteria from microcapsules without GT (B: 9.24 log CFU/mL, I: 9.34 log CFU/mL) to microcapsules with 5% GT (B: 9.39 log CFU/mL, I: 9.28 log CFU/mL) and 10% GT (B: 9.43 log CFU/mL, I: 9.36 log CFU/mL), respectively.

**Table 2:** Size, encapsulation yield and viability of bacteria in different beads containing green tea

**Beads Type**	**Beads Size (μm; n = 50)**	**Encapsulation yield (%; n = 15)**	**Viability (log CFU/mL, n = 4)**
I + 5% GT	344.19 ± 1	37.14 ± 0.4	9.28 ± 0.3
I + 10% GT	339.1.6 ± 1	35.33 ± 0.7	9.36 ± 0.2
B + 5% GT	329.36 ± 1	37.18 ± 0.4	9.39 ± 0.3
B +10% GT	331.25 ± 1	36.15 ± 0.7	9.43 ± 0.4
I	325.14 ± 0.5	38.24 ±0.5	9.34 ± 0.3
B	321.08 ± 0.9	37.24 ±0.8	9.24 ± 0.4
10% GT	318.23 ± 1		

### Survival of free and microencapsulated cells in SGJ

Several studies have shown that only microencapsulated probiotics were able to maintain viability in gastrointestinal conditions [20]. Immobilization of bacteria in alginate beads has previously been tested for improving the viability of probiotic bacteria in simulated gastric conditions [21]. Sultana et al. [4] found the encapsulation of bacteria in alginate beads did not effectively protect the organism from high acidity. On the other hand, some authors reported the effect of alginate encapsulation on survival of lactic bacteria in simulated gastrointestinal conditions [4,18], there is no uniformity in the reported results.

**Figure 3:**
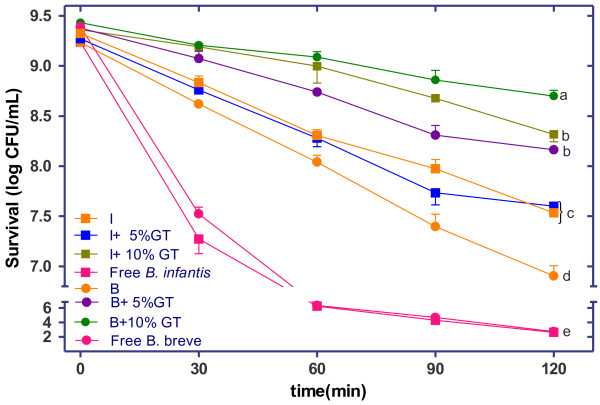
**Survival of free and encapsulated*B. infantis*(I) and*B. breve*(B) with and without addition of 5% and 10% green tea (GT) during exposure to simulated gastric juice at 37**°**C for 120 min.** The error bars indicate standard deviations from the mean values of three replicated experiments. Means with different letter in a column are significantly different (p < 0.05). For abbreviations see Table 4.

Viability of immobilized and free cells of probiotic bacteria with and without addition of GT, in simulated gastric juice was evaluated and the results were shown in Figure 3. Encapsulation in chitosan-coated alginate beads significantly (P < 0.05) protected survival of *B. infantis* and *B. breve*. Microencapsulated *B. infantis* and *B. breve* with or without GT were resistant to simulated gastric conditions. In capsules without addition of GT, the survival rate of bacteria was lower comparing with the capsules containing 5% and 10% GT addition. Thus, the survivability rate increased proportionally with the concentration of GT addition. A significant differences (P < 0.05) was noted between cell survival of *B. breve* with addition of 10% GT *vs*. all trials. Our results suggested that microcapsules with 5% and 10% GT extract increased the number of survival cells after 120 min of exposure to SGJ in comparation with microcapsules without GT addition. Thus, green tea exerts the stimulative effect on *B. infantis* and *B. breve*. It is estimated that 10^7^ CFU/mL of live probiotic cells are needed to confer health benefits to the consumer [22]. However, there was a rapid loss of free probiotic bacteria in SGJ, initial number of 9 log CFU/mL for free strains decreased to less than 2.6 log CFU/mL after exposure of 2 h.

Some reports have indicated differences among strains of probiotic bacteria with respect to their survival in acid environment [23]. Krasaekoopt et al. [5] found that encapsulation with alginate coated with chitosan was the best treatment to protect studied bacteria for all conditions tested. Molan et al. [24] demonstrated the prebiotic effect of green tea containing selenium promoting the growth of *Lactobacillus ssp.* and *Bifidobacterium ssp*. under *in vitro* conditions. The mechanism by which tea extract increased the growth of probiotic bacteria remain unclear, a possible partial explanation for this enhancing effect was presented by Molan et al. [24] consisting on the ability of polyphenols in green tea, to act as antioxidant and antiradical agents, to modulate the oxidative stress in the medium generated by the metabolic activities and consequently provide a better environment for the growth and multiplication of strains.

### Survival of microencapsulated probiotic bacteria in SIJ

Chitosan-coated alginate beads were the most effective in protecting probiotic bacteria from bile salt [19]. The chitosan coating provides protection in bile salt solution because an ion exchange reaction takes place when the beads absorb bile salt [25]. Krasaekoopt et al. [5] found that microencapsulation with alginate coated with chitosan was the best treatment to protect studied probiotic bacteria for all condition tested.

Viability of immobilized of free cells and *B. infantis* and *B. breve* with and without addition of (5%, 10%) GT in simulated intestinal juice was evaluated and the results were shown in Table 3. In the case of free *B. infantis* and *B. breve*, the initial average viable count of 9.24 log CFU/mL and 9.34 log CFU/mL was reduced to 4.56 log CFU/mL and 4.75 log CFU/mL after 90 min and the average viable number was further reduced to 2.89 log CFU/mL and 2.45 log CFU/mL after 120 min. The survival percentage of microencapsulated bacteria after exposure to SIJ for 120 min was highest in trials with 10% GT being 91.54% and 92.33% from the initial cell population found in encapsulated I + GT 10% and B + GT 10%. 10% GT exert a positive effect on survivability of probiotic bacteria after 120 min of SIJ exposure, enhancing the number of *B. infantis* with 5.05% and *B. breve* with 7.38% when was coencapsulated with them.

**Table 3:** **Number of survival cells (log CFU/mL) during sequential incubation (37**°**C) in simulated intestinal juice**

**Beads Type**	**Simulated Intestinal Juice (SIJ)**			
	0 min	60 min	90 min	120 min
I +5% GT	8.85 ± 0.3	8.75 ± 0.4	8.20 ± 0.5	7.89 ± 0.6
I +10% GT	8.99 ± 0.1	8.79 ± 0.1	8.48 ± 0.6	8.23 ± 0.4
B + 5%GT	8.98 ± 0.5	8.64 ± 0.6	8.44 ± 0.1	7.46 ± 0.8
B + 10%GT	8.87 ± 0.7	8.75 ± 0.9	8.68 ± 0.5	8.19 ± 0.5
B	9.04 ± 0.3	8.72 ± 0.5	8.03 ± 0.4	7.68 ± 0.7
I	9.11 ± 0.6	8.84 ± 0.1	8.15 ± 0.7	7.88 ± 0.6
Free *B. breve*	9.24 ± 0.2	6.81 ± 0.8	4.75 ± 0.5	2.45 ± 0.4
Free *B. infantis*	9.34 ± 0.1	6.93 ± 0.1	4.56 ± 0.2	2.89 ± 0.3

### Resistance to refrigerated storage

**Figure 4:**
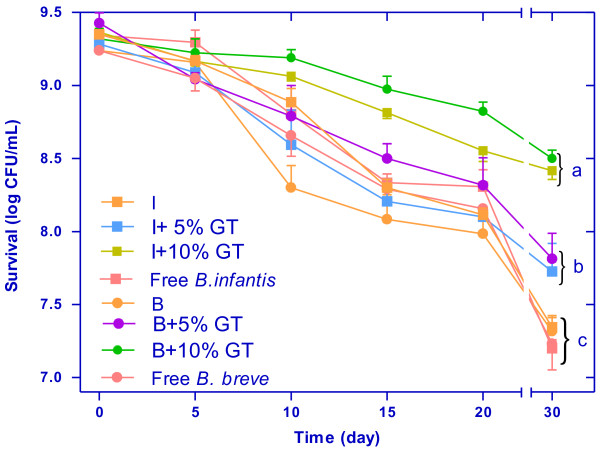
**Survival of*B. infantis*(I) and*B. breve*(B) with and without addition of 5% and 10% green tea (GT) under refrigerated storage (4 ± 1**°**C).** Means (n = 3) ± SD. Means with different letter in a column are significantly different (p < 0.05). For abbreviations see Table 4.

Experiments were performed in order to evaluate the efficiency of immobilization treatment for increasing the probiotics viability under refrigeration. The results of the *B. infantis* and *B. breve* survival of with and without addition of green tea extract under 30 days of incubation at 4 ± 1°C are presented in Figure 4. Results showed that at the end of this time, the immobilized cells in 10% GT had the lowest loss of viability (0.94 log CFU/mL for *B. infantis* and 0.82 log CFU/mL for *B. brevis*). The viability of microencapsulated cells showed different stability between microcapsules with or without GT in the same storage conditions. After 30 days, the survival of free *B. infantis* and *B. breve* decreased from 3.5 x 10^9^ to 3.4 x 10^7^ CFU/mL and from 2.4 x 10^9^ to 3.1 x 10^7^ CFU/mL respectively. The number of microencapsulated bacteria with 10%, 5% GT was significantly higher (P < 0.05) than microencapsulated bacteria without GT and free bacteria. The rate of decrease was significantly different (P < 0.05) between the microencapsulated with and without 10% GT. Koo et al. [26] reported that probiotic bacteria loaded in chitosan-coated alginate microcapsules showed higher storage stability than free cell culture. We also observed a similar effect in our study. One of the properties for a given microorganism to be considered probiotic is its capacity to survive storage as a formulated product. In general, fermented products containing added probiotics should be stored under refrigeration at 4°C. Our results, suggest that immobilization of *B. infantis* and *B. breve* with GT improve viability of probiotics during refrigeration storage

## Conclusions

The results of this study clearly show that the major compounds in green tea are epigallocatechin gallate followed by caffeine and epigallocatechin. 5%, 10% green tea coencapsulated in chitosan coated alginate beads, exert a stimulative effect on *B. infantis* and *B. breve*. The microencapsulation with 10% green tea (w/v) was more effective in maintaining the bacteria stability and increased their viability by storage at refrigeration temperature during 30 days. Also, green tea, significantly improved the bacterial survival in simulated gastrointestinal environment, and allows viable cells reach a beneficial level of probiotic. In conclusions, green tea microcapsules with probiotic bacteria offers an effective way to increase the life-spam and survivability in simulated gastrointestinal juices and maintaining their survival during refrigerated storage. For a health perspective, the results confirm the growing interest in probiotic bacteria and the perceived benefit of increasing their numbers in the gastrointestinal tract.

## Methods

### Preparation of tea extracts

Green tea (GT) was purchased from an online shop and is presently available on the market. GT is originally from China and the content of total polyphenols was reported on the prospectus as 40%. The aqueous extracts were made by adding 10 ml water (100°C) to 0.1 g or 0.2 g tea leaves and brewing for 10 min with stirring and removing solid matter by filtration.

### Chemical characterization of green tea extract

The chromatographic system used was an HPLC-DAD Agillent Technology (USA) series 1200 coupled with LC/MS single-quadrupole mass spectrometer equipped with a pneumatically assisted ESI source. The column was a Eclipse XDB-C18, 150x4,6 mm, **(5** μm) from Agillent (USA). The phenols were separated with a mobile phase consisting of 1% (v/v) formic acid (mobile phase A) and acetonitril (mobile phase B). A gradient run was started at 90% gradient A, decreasing in 30 min to 75%, further decreasing to 10% in 15 min and then back to 90% in 10 min. Total run for each sample was 55 min. The flow rate was 0.5 ml/min. The diode array detector was set to acquisition range of 200-600 nm. The HPLC effluent entered the mass spectrometer through an electrospray capillary set at 3.0 kV at a source block temperature of 100°C and a desolvation gas temperature of 350°C. Nitrogen was used at flow rate of approximately 8 L/min. The mass spectra between *m/z* 100 and 600, were obtained at a scan speed of 250 *m/z*.

### Bacterial strains and culture condition

*Bifidobacterium infantis* ATCC 15697 and *Bifidobacterium breve* ATCC 15700 were purchased in lyophilized form Bioaqua, Romania. Bacteria were routinely grown in MRS broth. Shortly, freeze-dried cells were inoculated into 5 mL MRS (de Man, Rogosa, Sharpe) broth (Merck, Germany) and incubated at 37°C, for 24 h under anaerobic conditions, and afterwards sub-cultured into 95 mL broth and incubated under the same conditions. The cells were harvested by centrifugation at 3000 g for 5 min at 4°C washed twice with sterile 0.9% (w/v) sodium chloride solution and resuspended in 2.5 mL of sodium chloride solution 0.5% (w/v).

### Microencapsulation and coating procedures

The method described by Sheu and Marshall [27] was adopted for microencapsulation of bacteria strains. The water-GT extract was mixed with 20 g/L of sodium alginate powder (Promova Biopolymer Norway), and sterilized. The cell suspension (2.1x10^9^ CFU/mL)were used as free cells or were aseptically mixed with 10 mL of 2% (w/v) alginate solution containing or not 5% or 10% Green Tea (GT) extract, (Table 4) and were applied to the immobilization system. The chitosan and sodium alginate solutions were prepared according to Krasaekoopt et al. [28]. Briefly, the beads were immersed in 100 mL of chitosan solution 0.4% (w/v) and shaken at 100 rpm, 37° C for 40 min on an orbital shaker for coating. The chitosan- coated alginate beads were collected by centrifugation (500 rpm, 10 min at 4° C). The microcapsules were washed twice with 0.9% (w/v) sodium chloride solution and ressuspended in 50 mL of 0.5% (w/v) sodium chloride solution.

**Table 4:** Chitosan coated alginate beads with bacteria and green tea

**Trials**	**Abbreviation**
Beads with 5% Green Tea and *B. infantis*	I +5% GT
Beads with 10% Green Tea and *B. infantis*	I +10% GT
Beads with 5% Green Tea and *B. breve*	B + 5% GT
Beads with 10% Green Tea and *B. breve*	B + 10% GT
Beads with *B. infantis*	I
Beads with *B. breve*	B
Beads with 10% Green Tea	10%GT

The trials abbreviations are presented.

### Survival assay and numeration of microencapsulated bacteria

Entrapped bacteria were released by homogenizing 1 mL of bead suspension in 9 mL of sodium citrate 0.1 M for 10 min, stirred diluted and poured in MRS agar plate. The plates were incubated 2 days at 37°C, and the released bacteria enumerated as CFU/mL. The encapsulation yield (EY), which is a combined measurement of the efficacy of entrapment and survival of viable cells during the microencapsulation procedure, was calculated as: *EY* = (N/N_o_) x 100. N is the number of free living cells released from the microcapsules, and N_o_ is the number of free cells added to the biopolymer mix during the production of microcapsules. The particle size and formation of microcapsules were measured with a light microscope (Axio, Observer A1, Zeiss). The data analysis was performed using software UTHSCSA (University of Texas Health Science Center, San Antonio) Image Tool software (University of Texas Health Science Center, San Antonio, TX, USA).

### Resistance to gastrointestinal conditions

Simulated gastric juice (SGJ) consisted of 9 g/L of sodium chloride containing 3 g/L of pepsin with pH adjusted to 2.0 with hydrochloric acid. 1 mL of cell suspension of *B. infantis* or *B. breve* were mixed in 9 mL SGJ and incubated for 30, 60, 90 and 120 min at 37°C with constant agitation of 50 rpm.

Simulated intestinal juice (SIJ) was prepared by dissolving bile salts (Oxoid, Basingstoke, UK) in intestinal solution (6.5 g/L NaCl, 0.835 g/L KCl, 0.22 g/L CaCl_2_ and 1.386 g/L NaHNO_3_ ) pH 7.5 to final concentrations of 3.0 g/L (Chavarri *et al*., 2010). Triplicate samples were mixed, incubated at 37°C and sampled 30, 60, 90, 120 min after addition of cell suspension. Surviving bacteria were numerated by pour plated counts in MRS agar incubated at 37°C.

### Resistance to refrigerated storage

The viability of probiotic bacteria under refrigeration was evaluated by incubating 1 mL (approximatively 3.8 x 10 ^9^ cells/mL) of free and immobilized cell suspension in 9 mL of 5% (w/v) sterile sodium chloride solution. Aliquots of 1 mL were taken every day for 30 days to determine the total number of viable cells.

### Statistical analysis

Results for 3 individual experiments were used to calculate the mean of cell counts. Analysis of variance (ANOVA) and Duncan’s multiple range tests were performed to analyze the results. Significance of difference was defined at the 5% level (P < 0.05). All statistical analysis was carried out using Graph Pad Version 4.0 (Graph Pad Software Inc; San Diego, CA, USA).

## Competing interests

The authors declare that they have no competing interests.

## Authors’ contributions

CS carried out the chemical composition of green tea extract taking into consideration the separation and quantification of extracts. DV contributed to the microencapsulation experimental work, exposure to simulated gastrointestinal juices and refrigerated storage. All authors read and approved the final manuscript.
